# Effects of digital health interventions on objectively measured physical activity during the perinatal period: a systematic review and meta-analysis

**DOI:** 10.3389/fpubh.2026.1786474

**Published:** 2026-04-02

**Authors:** Yang Xu, Mingyu Liao, MeiLu Zhang

**Affiliations:** 1 College of Aviation Physical Education, Civil Aviation Flight University of China, Chengdu, China; 2 School of Humanities Education and Application, Sichuan Technology and Business University, Chengdu, China

**Keywords:** daily step count, digital health interventions, meta-analysis, mHealth, perinatal period, physical activity

## Abstract

**Objectives:**

Insufficient physical activity is prevalent among perinatal women, and digital health interventions offer a promising avenue to promote engagement in physical activity within this population. However, previous studies have relied heavily on self-reported data, lacking a systematic synthesis based on objective measurements. This study aims to systematically evaluate the effects of digital health interventions on objectively measured physical activity and sedentary behavior in perinatal women.

**Methods:**

A systematic search was conducted in PubMed, Embase, Web of Science, and the Cochrane Library databases from inception to December 20, 2025. Fourteen randomized controlled trials (RCTs) involving 2,101 participants were included. The Risk of Bias 2.0 (RoB 2.0) tool was used to assess bias risk, random-effects models were employed to pool effect sizes, and the quality of evidence was evaluated using the GRADE system.

**Results:**

The meta-analysis showed that, following the exclusion of outliers via sensitivity analysis, digital health interventions significantly increased daily step counts (MD = 0.68, *p* < 0.00001, *I*^2^ = 0%; moderate quality of evidence) and light physical activity (LPA; MD = 13.04 min/day, *p* = 0.03, *I*^2^ = 0%; low quality of evidence) in perinatal women. However, the interventions did not yield significant improvements in moderate-to-vigorous physical activity (MVPA; MD = 1.37 min/day, *p* = 0.16) or sedentary time (MD = −1.70 min/day, *p* = 0.79), with extremely high consistency observed across studies (*I*^2^ = 0%). Subgroup analyses indicated that the effect on increasing step counts remained highly consistent across different perinatal stages and intervention modes.

**Conclusion:**

Digital health interventions can effectively and robustly enhance daily baseline activity levels in perinatal women, with the observed increments potentially reaching the minimal effective dose for improving metabolic health. However, current intervention designs face challenges in driving high-intensity behavior change and disrupting sedentary habits. Future research should explore more targeted and personalized intervention strategies.

**Systematic review registration:**

This systematic review and meta-analysis has been registered in PROSPERO (www.crd.york.ac.uk/prospero), identifier CRD420261280936.

## 1 Introduction

The perinatal period, encompassing pregnancy and the first year postpartum, represents a distinct phase characterized by profound physiological and psychological changes in women, serving as a critical window for establishing long-term health behaviors (1). However, due to increased physiological burdens, parenting pressures, and time fragmentation, a significant proportion of perinatal women globally exhibit insufficient levels of physical activity (PA) (2, 3). Evidence indicates that physical inactivity during the perinatal period is closely associated with a range of adverse health outcomes, including gestational diabetes mellitus (GDM), hypertensive disorders of pregnancy (HDP), excessive gestational weight gain, and postpartum depression (4, 5). Conversely, maintaining moderate physical activity not only contributes to improving metabolic health for both mother and infant (6) but also effectively alleviates postpartum stress and enhances quality of life (7, 8). Consequently, identifying low-cost, high-compliance intervention models to promote physical activity engagement among perinatal women has become a focal point in public health.

With the rapid advancement of information technology, Digital Health Interventions (DHIs) have demonstrated potential advantages in behavioral medicine management (9). DHIs encompass various modalities, including smartphone applications (Apps), short message services (SMS), remote health coaching, and feedback from wearable devices (10). Compared to traditional face-to-face counseling, digital health protocols offer core advantages such as high accessibility, real-time feedback, convenience, and freedom from spatiotemporal constraints, thereby better aligning with the fragmented lifestyle patterns of perinatal women (11). Currently, several systematic reviews—synthesizing evidence from approximately 11 to 16 primary studies each—have summarized the impact of digital health interventions on perinatal physical activity, yet the conclusions remain notably inconsistent. For instance, one systematic review of 15 randomized controlled trials (RCTs) focusing on pregnant women indicated that mobile Apps and telephone-based interventions significantly increased self-reported physical activity levels (12). In contrast, a meta-analysis specifically examining 11 exclusively digital interventions reached a divergent conclusion, suggesting there is currently insufficient evidence to support the efficacy of such methods in effectively improving maternal physical activity or managing gestational weight (13). Furthermore, even among reviews reporting positive outcomes, discrepancies persist. Existing meta-analyses which include a recent synthesis of 16 studies with 10 included in the quantitative meta-analysis have noted that while digital health technologies may yield moderate improvements in total activity volume, the effect sizes for moderate-to-vigorous physical activity remain weak, with significant clinical heterogeneity observed across studies (14).

Despite the accumulation of research regarding digital health interventions in this domain, significant limitations remain. First, while previous systematic reviews have synthesized up to 18 RCTs, they have either relied heavily on self-reported data or are limited by literature searches conducted only up to 2022 (15). Given that perinatal women are susceptible to recall bias or social desirability bias, self-reported activity levels often deviate from actual behaviors (16). Second, although evidence from cohort studies indicates that pregnant women exhibit stable sedentary behavior of nearly 10 h per day which is associated with increased odds of adverse pregnancy outcomes, there is still a lack of systematic synthesis incorporating the most recent evidence through late 2025 regarding objectively measured physical activity across different intensities, specifically light physical activity versus moderate-to-vigorous physical activity and sedentary behavior (17).

Therefore, this study aims to comprehensively and objectively evaluate the effects of digital health interventions on objectively measured daily step counts, LPA, MVPA, and sedentary time in perinatal women through a systematic review and meta-analysis of randomized controlled trials. By quantitatively synthesizing objective data and employing GRADE certainty assessments, this study seeks to clarify the true effects of digital health interventions on distinct behavioral metrics and explore their generalizability across different delivery modes. The findings are expected to not only enrich the evidence base for perinatal exercise interventions but also provide a reference for decision-making in optimizing the allocation of clinical maternal and child health resources and the development of digital health protocols.

## 2 Methods

This study protocol has been registered in PROSPERO (Registration number: CRD420261280936) and was conducted and reported strictly following the Preferred Reporting Items for Systematic Reviews and Meta-Analyses (PRISMA 2020) checklist (18).

### 2.1 Search strategy

A systematic search was conducted in PubMed, Embase, CINAHL, Web of Science, and the Cochrane Library from their inception to December 20, 2025. The search strategy employed a combination of subject headings (e.g., MeSH terms) and free-text terms, structured around four core concepts: (1) the perinatal population (e.g., “pregnancy,” “postpartum,” “maternal,” “parity”); (2) digital health interventions (e.g., “digital health,” “mHealth,” “mobile app,” “telehealth,” “wearable devices”); (3) physical activity and sedentary behavior indicators (e.g., “physical activity,” “exercise,” “daily steps,” “sedentary behavior”); and (4) randomized controlled trials (e.g., “randomized controlled trial”, “RCT”). Search queries were adapted to the syntax of each database, and the complete search strings are provided in Appendix Text 1. Additionally, to ensure comprehensiveness, we manually screened the reference lists of the included studies and relevant systematic reviews to identify any potentially eligible articles that may have been missed.

### 2.2 Inclusion and exclusion criteria

Inclusion criteria were strictly formulated based on the PICOS framework. Regarding Participants (P), we included women in the perinatal period, which was operationally defined as the duration from the onset of pregnancy through one year postpartum. This population encompassed both general cohorts and individuals with high-risk conditions such as GDM or HDP, regardless of age, ethnicity, or BMI. Regarding Interventions (I), eligible studies utilized structured behavioral programs centered on digital health technologies including smartphone apps, SMS, or wearable feedback. These protocols were required to integrate specific behavior change techniques (BCTs) grounded in health behavior theories to systematically modify activity patterns. Regarding Comparators (C), control groups received standard care, printed materials, or technologies lacking active feedback. Regarding Outcomes (O), studies must have reported at least one objective physical activity metric including daily steps, MVPA, LPA, or sedentary time, recorded via sensors. Finally, Study Design (S) was restricted to randomized controlled trials (RCTs). Studies were excluded if they met any of the following criteria: (1) non-RCT designs including quasi-experimental studies or protocols; (2) participants with severe complications contraindicating physical activity; (3) digital protocols lacking interactive behavioral feedback; (4) reliance on self-reported data; or (5) conference abstracts and editorials.

### 2.3 Data collection

Data extraction was performed independently by two investigators using a pre-designed, standardized data extraction form. Any discrepancies arising during the process were resolved through discussion and re-examination of the original literature, with a third senior investigator consulted to reach consensus when necessary. The extracted data were strictly categorized according to the PICOS framework: (1) General characteristics: first author, publication year, country, study phase (pregnancy or postpartum), and study design; (2) Participants (P): sample size (number randomized vs. analyzed), mean age, and mean Body Mass Index (BMI); (3) Interventions (I): specific components of the digital health intervention (e.g., smartphone apps, SMS, video calls), implemented behavior change techniques (BCTs), and intervention duration; (4) Comparators (C): detailed protocols for standard prenatal/postpartum care or other control conditions; and (5) Outcomes (O): objective measurement device specifications (brand and model), wear criteria (e.g., required daily wear time and valid days), device placement (e.g., wrist, hip, or thigh), and the means and standard deviations (SD) for physical activity metrics (daily steps, MVPA duration, sedentary time, and LPA). For key effect sizes reported as medians, interquartile ranges, confidence intervals, or *p*-values, data were converted using statistical formulas recommended by the Cochrane Handbook. In cases of missing or conflicting data, attempts were made to contact the corresponding authors via email to obtain the original data, ensuring the accuracy and transparency of the meta-analysis.

### 2.4 Risk of bias and certainty of evidence

The methodological quality of the included studies was assessed using the Risk of Bias 2 (RoB 2) tool for randomized controlled trials, as recommended by the Cochrane Collaboration (19). The assessment covered five specific domains: bias arising from the randomization process, bias due to deviations from intended interventions, bias due to missing outcome data, bias in measurement of the outcome, and bias in selection of the reported result. Each domain was classified as having “low risk,” “some concerns,” or “high risk” of bias, culminating in an overall risk of bias judgment for each study. The certainty of the evidence was evaluated using the Grading of Recommendations Assessment, Development and Evaluation (GRADE) system (20). Evidence quality was assessed for potential downgrading based on five factors: risk of bias, inconsistency, indirectness, imprecision, and publication bias, with the quality ultimately categorized as high, moderate, low, or very low. Two reviewers independently performed the quality assessments; discrepancies were resolved through discussion to reach a consensus or by consulting a third expert if necessary.

### 2.5 Data analysis

Statistical analyses were performed using Review Manager (RevMan) version 5.4. Given the inherent clinical and methodological heterogeneity regarding participant characteristics (e.g., perinatal stage, baseline BMI), digital health intervention components, and follow-up duration, a random-effects model was applied *a priori* for the synthesis of effect sizes. This approach is considered more conservative than a fixed-effects model, as it accounts for both within-study and between-study variations to yield more robust estimates. The primary outcomes (daily step counts, minutes of LPA, minutes of MVPA, and sedentary time) were all continuous variables. The mean difference (MD) with 95% confidence intervals (CI) was calculated when measurement units were consistent; otherwise, the standardized mean difference (SMD) was utilized to pool data across studies employing different measurement tools or reporting units. Heterogeneity was quantitatively assessed using the Cochrane *Q* test and the *I*^2^ statistic, where *I*^2^ ≤ 50% indicated acceptable heterogeneity and *I*^2^ > 50% indicated significant heterogeneity. To investigate potential sources of significant heterogeneity, subgroup analyses (stratified by perinatal stage or intervention mode) and sensitivity analyses (using the leave-one-out method) were conducted. Furthermore, where data permitted, random-effects dose–response models were employed to evaluate potential linear or non-linear associations between intervention dose and improvements in physical activity. Finally, publication bias was assessed using funnel plots combined with Egger’s test, with statistical significance established at *p* < 0.05.

## 3 Results

### 3.1 Study selection

**Figure 1:**
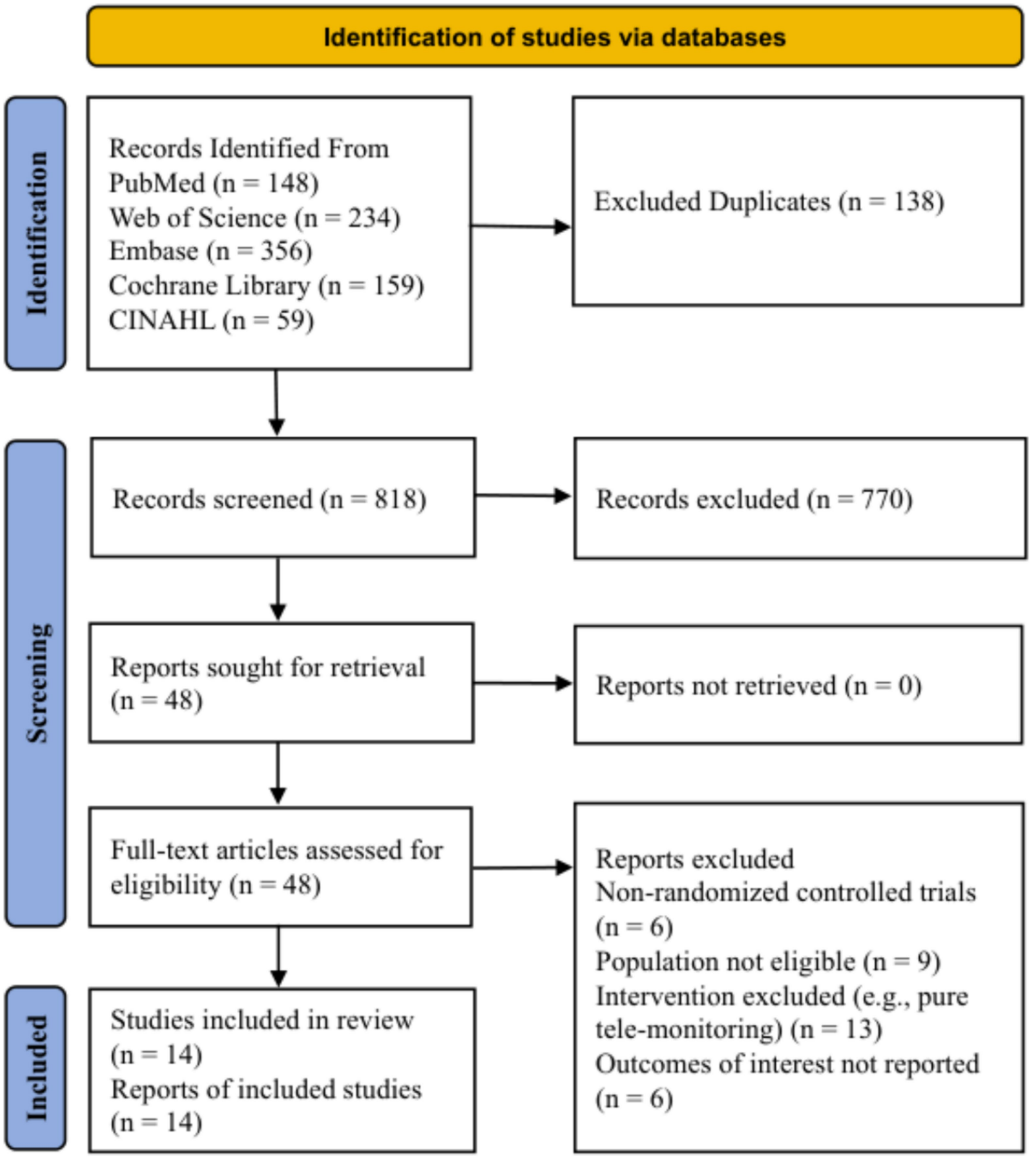
Preferred reporting items for systematic reviews and meta-analysis (PRISMA) study flow diagram.

A total of 956 records were identified through the initial database search, including 148 from PubMed, 234 from Web of Science, 356 from Embase, 159 from the Cochrane Library, and 59 from CINAHL. After removing 138 duplicates using EndNote software via both automatic and manual verification, 818 records remained for title and abstract screening. Following the preliminary screening, 770 records were excluded for clearly not meeting the inclusion criteria, and the full texts of the remaining 48 articles were retrieved for detailed eligibility assessment. During the full-text review phase, 34 studies were excluded for the following reasons: (1) inappropriate study design (*n* = 6), consisting of non-randomized controlled trials; (2) ineligible study population (*n* = 9), primarily due to the inclusion of high-risk pregnancy cohorts with severe complications (e.g., placenta previa, preeclampsia) rather than the general healthy population defined in this study; (3) ineligible interventions (*n* = 13), where protocols did not qualify as digital health interventions (e.g., pure remote monitoring lacking interactive feedback components); and (4) ineligible outcomes (*n* = 6), characterized by the absence of objective data recording or failure to report the relevant indicators of interest. Ultimately, 14 randomized controlled trials were included in the systematic review and meta-analysis. The literature screening process is illustrated in Figure 1.

### 3.2 Study characteristics

A total of 14 randomized controlled trials (RCTs) involving 2,101 randomized participants were ultimately included in this study (21–34) (Table 1). The studies were conducted across eight countries: the United States (*n* = 6) (21, 22, 27, 28, 32, 34), Australia (*n* = 2) (23, 24), Denmark (29), Sweden (26), Germany (31), France (30), Israel (25), and China (33). Regarding the perinatal phase, 10 studies focused on the pregnancy period (21–23, 26, 27, 29–34), three targeted the postpartum phase (24, 25, 28), and one study spanned the entire transition from pregnancy through the postpartum period (27). Sample sizes ranged from 30 to 351 participants (21, 34). The mean age of participants ranged from 27.0 to 34.5 years (24, 34), with baseline mean Body Mass Index (BMI) ranging from 23.9 kg/m^2^ to 32.1 kg/m^2^ (28, 31).

**Table 1:** Characteristics of the included studies.

Study ID (Author, Year)	Phase	N(Initial/Analyzed)	Participant characteristics (Age, BMI)	Intervention protocol (mHealth components and BCTs)	Control condition	Intervention timing and duration	Objective measurement (device, placement, criteria)	Outcomes
Choi et al. (21)	Pregnancy	30/29	33.7 ± 2.6 y; BMI 27.7 ± 3.7	Mobile App + Fitbit feedback; goal setting, daily SMS reminders, self-monitoring	Fitbit wear only (attention control)	12 weeks (From ~19w to ~31w gestation)	Fitbit Ultra; Not specified; Valid wear: ≥ 1,000 steps/day	Steps
Cheung et al. (24)	Postpartum	60/31	34 ± 4 y; BMI 29.0 ± 8.2	SMS + Fitbit Flex; focused on motivational feedback and adaptive step goals	Postpartum usual care + Fitbit (no feedback)	Up to 38 weeks (From ~4.3 m to ~13.5 m postpartum)	Fitbit Flex®; Wrist; Excluded if <1% days recorded	Steps
Hayman et al. (23)	Pregnancy	77/77	Mean 29 y; Mean BMI 26	Web-based tailored intervention (Fit4Two); personalized feedback, SMART goals, action planning	Library access only	4 weeks (From ~22w to ~26w gestation)	GeneActiv; Non-dominant wrist; ≥4 days (incl. weekend), ≥10 h/day	MVPA
Lewey et al. (28)	Postpartum	127/127	32.3 ± 5.6 y; BMI 32.1 ± 7.5	Gamified text + Fitbit; virtual team competition, points system, social incentives	Fitbit + daily goal achievement SMS	12 weeks (From ~3.6 m to ~6.6 m postpartum)	Fitbit Inspire HR; Not specified; Valid wear: ≥ 1,000 steps/day	Steps
Knudsen et al. (29)	Pregnancy	220/215	31.9 ± 4.4 y; BMI 24.2 ± 3.7	App + Motivational counseling; self-monitoring, real-time feedback, Facebook community support	Standard prenatal care	~27 weeks (From ~13w gestation until delivery)	SENS motion; Lateral thigh; ≥22 h/day, ≥3 valid days	MVPA, LPA, Sed
Cabre et al. (34)	Pregnancy	351/178	27.0 ± 6.0 y; BMI 28.5 ± 5.8	Multicomponent App + Scale + Fitbit + Coaching feedback + Facebook group	Standard WIC nutrition education	~25 weeks (From ~15w gestation until delivery)	ActiGraph GT3X+; Non-dominant wrist; ≥3 days, ≥10 h/day	Steps, MVPA, LPA, Sed
Ganer Herman et al. (25)	Postpartum	215/215	32.8 ± 5.3 y; BMI 27.9 ± 5.8	Digital pedometer with personalized feedback (at 8, 24, and 48 h post-surgery)	Routine post-Cesarean care	48 h (Starting immediately post-surgery)	Digital Pedometer; Wrist; Continuous monitoring for 48 h post-surgery	Steps
Muller et al. (30)	Pregnancy	250/59	29.6 ± 4.5 y; BMI 24.5 ± 4.8	“Bouge Grossesse” App; personalized coaching, self-monitoring, push notifications	Placebo App (displaying steps only)	12 weeks (From ~18w to ~30w gestation)	Smartphone internal sensor; Not specified; Analyzed rate of change in step counts	Steps
Tinius et al. (27)	Preg/Post	38/35	18–44 y; BMI 29.0 ± 7.5	BumptUp® App; progress tracking, exercise videos, social support, symptom monitoring	Evidence-based printed brochure	~24 weeks (From ~16w gestation to 12w postpartum)	ActiGraph wGT3X-BT; Wrist; 30 Hz sampling; Freedson cut-points	MVPA, LPA, Sed
Li et al. (33)	Pregnancy	200/178	30.2 ± 4.0 y; BMI 27.9 ± 3.3	WeChat Official Account + Fitness Band; diet/step goals, personalized feedback, telephone follow-up	Routine hospital prenatal care	~16 weeks (From ~14w to ~30w gestation)	Fitness Band; Not specified; Target: 6,000 steps/day + 150 min/week brisk walking	Steps
Huberty et al. (22)	Pregnancy	80/50	29.75 ± 4.4 y; BMI 26.86 ± 5.9	Text4baby SMS intervention; exercise reminders, motivational support, goal setting	Standard Text4baby (general health SMS)	8 weeks (From ~18w to ~26w gestation)	ActiGraph GT3X+; Right hip; ≥4 days, ≥10 h/day	MVPA, Sed
Gibbs et al. (32)	Pregnancy	51/47	Mean 32 y; Mean BMI 28	Remote coaching + Fitbit/AppleWatch + Standing desk + Facebook group	No-contact control	~21 weeks (From ~19w gestation until delivery)	activPAL3; Anterior thigh; 24 h continuous wear, ≥5 valid days	Steps, MVPA, LPA, Sed
Sandborg et al. (26)	Pregnancy	305/272	31.5 ± 4.3 y; BMI 24.5 ± 4.0	HealthyMoms App; diet/exercise monitoring, personalized feedback, push notifications	Standard Swedish prenatal care	18 weeks (From ~14w to ~32w gestation)	ActiGraph wGT3X-BT; Non-dominant wrist; ≥4 days, ≥10 h/day	MVPA, Sed
Téoule et al. (31)	Pregnancy	97/89	33.6 ± 3.8 y; BMI 23.9 ± 3.6	Online video health coaching; motivational interviewing, personalized support, progress feedback	Standard prenatal care	~26 weeks (From ~14w gestation until delivery)	Objective pedometer; Not specified; Total daily steps recorded	Steps

Data are presented as Mean ± Standard Deviation (SD) unless otherwise specified. All included studies utilized objective hardware devices for measurement. Valid data were generally defined as ≥10 h of wear time during waking hours for ≥4 days per week. Intervention protocols incorporated Behavior Change Techniques (BCTs), primarily comprising goal setting, self-monitoring, feedback, and motivational interviewing. N (Initial/Analyzed) denotes the total number of participants randomized versus the number included in the final statistical analysis, intended to transparently reflect participant attrition. BMI, Body Mass Index; RCT, Randomized Controlled Trial; mHealth, mobile health; GDM, Gestational Diabetes Mellitus; HDP, Hypertensive Disorders of Pregnancy; NR, Not Reported; Steps, daily step count; MVPA, Moderate-to-Vigorous Physical Activity; LPA, Light Physical Activity; Sed, Sedentary Time.

Regarding intervention delivery, the intervention groups received support via mobile health (mHealth) technologies. Specific protocols included smartphone applications (*n* = 6) (21, 26, 27, 29, 30, 34), text-based short message services (SMS) (*n* = 3) (22, 24, 28), and remote personal health coaching integrated with video calls (*n* = 5) (23, 25, 31–33). Core behavior change techniques (BCTs) implemented during the interventions included goal setting (e.g., SMART goals or step targets) (23), self-monitoring (21), personalized automated feedback (25), social support (27), gamification, and environmental restructuring (e.g., provision of standing desks) (28). Control group conditions encompassed standard prenatal/postpartum care (29), provision of objective measurement devices without feedback features (24), printed educational materials (27), or simple health education devoid of active intervention components (30). Intervention durations varied widely, ranging from short-term monitoring of 48 h immediately post-surgery to long-term interventions lasting up to 38 weeks (24, 25). All studies mandated the use of objective hardware devices for measurement. Twelve studies reported daily step counts, primarily recorded via Fitbits or built-in smartphone pedometers, while MVPA (eight studies), sedentary time (six studies), and light physical activity (five studies) were captured primarily using clinical-grade accelerometers such as ActiGraph (34), activPAL (32), GeneActiv (23), and SENS motion (29). Measurement criteria were stringent; the majority of studies required a valid wear time of 3–5 days (32, 34), defined as ≥ 10 h of wear during waking hours or 24-h continuous monitoring (28, 32).

### 3.3 Risk of bias

**Figure 2:**
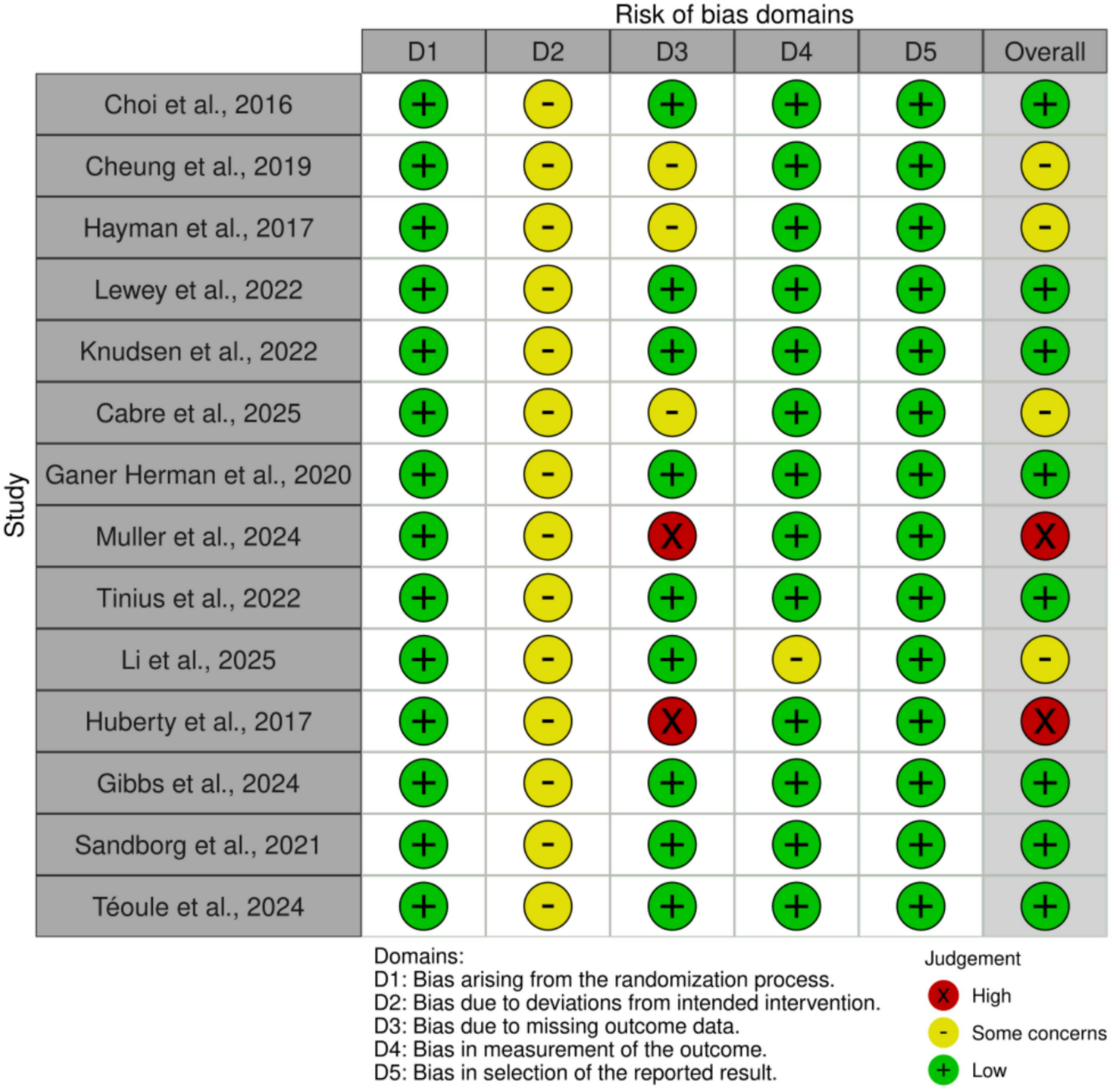
Risk of bias summary: review of the authors judgments about each risk of bias item for each included study.

**Figure 3:**
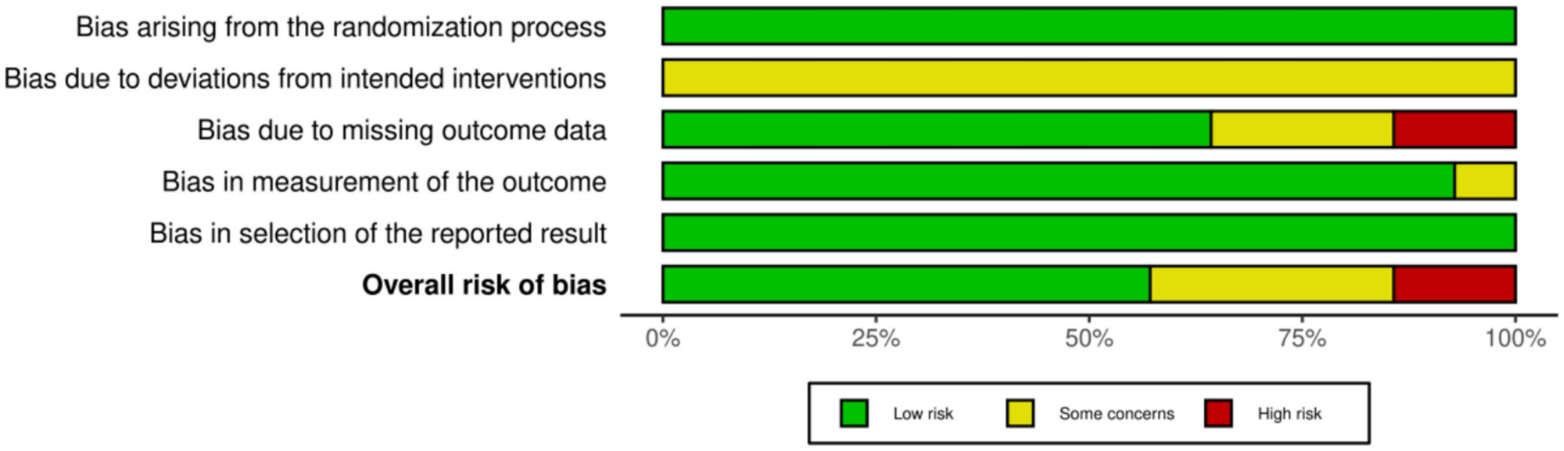
Risk of bias graph: review authors’ judgments about each risk of bias item, presented as percentage of included studies.

The risk of bias in the 14 included randomized controlled trials was assessed using the Cochrane Risk of Bias tool 2.0 (RoB 2.0) standards (Figures 2, 3). Regarding specific domains, all included studies (*n* = 14) demonstrated a low risk of bias arising from the randomization process (D1) and selection of the reported result (D5). These studies provided clear descriptions of random sequence generation, ensured allocation concealment, and reported outcomes consistent with pre-specified protocols. Due to the inherent nature of exercise interventions and mHealth technologies, all 14 studies were classified as raising “some concerns” regarding deviations from intended interventions (D2), primarily attributed to the inability to blind participants and personnel. Regarding bias due to missing outcome data (D3), Muller (2024) and Huberty (2017) were judged as “high risk” due to extremely high attrition rates (completion rates of only 13–34%) and substantial loss of objective accelerometer data (37.5%) without valid imputation, respectively (22, 30). Additionally, Cheung (2022), Hayman (2017), and Cabre (2025) were rated as raising “some concerns” as they either restricted analysis to samples with complete data or exhibited elevated rates of loss to follow-up (23, 30, 34). In terms of measurement of the outcome (D4), with the exception of Li et al. (33)—which was classified as raising “some concerns” due to partial reliance on self-reported questionnaire data for exercise assessment—the remaining studies were deemed low risk as they relied exclusively on data exported from objective devices.

The comprehensive evaluation indicates that the overall methodological quality of the included literature is robust. Based on the overall risk assessment, 10 studies were categorized as having “low risk” or “some concerns.” The primary drivers for downgraded ratings were the unavoidable lack of blinding in behavioral interventions and data loss associated with compliance in wearing wearable devices during long-term interventions. Although two studies (Muller, 2024; Huberty, 2017) were judged as “high risk” due to data integrity issues (22, 30), the evidence base of this review maintains high validity and robustness, given the excellent performance of the majority of studies regarding randomization, objective measurement, and reporting standards.

### 3.4 Meta-analysis results

#### 3.4.1 Daily steps

**Figure 4:**
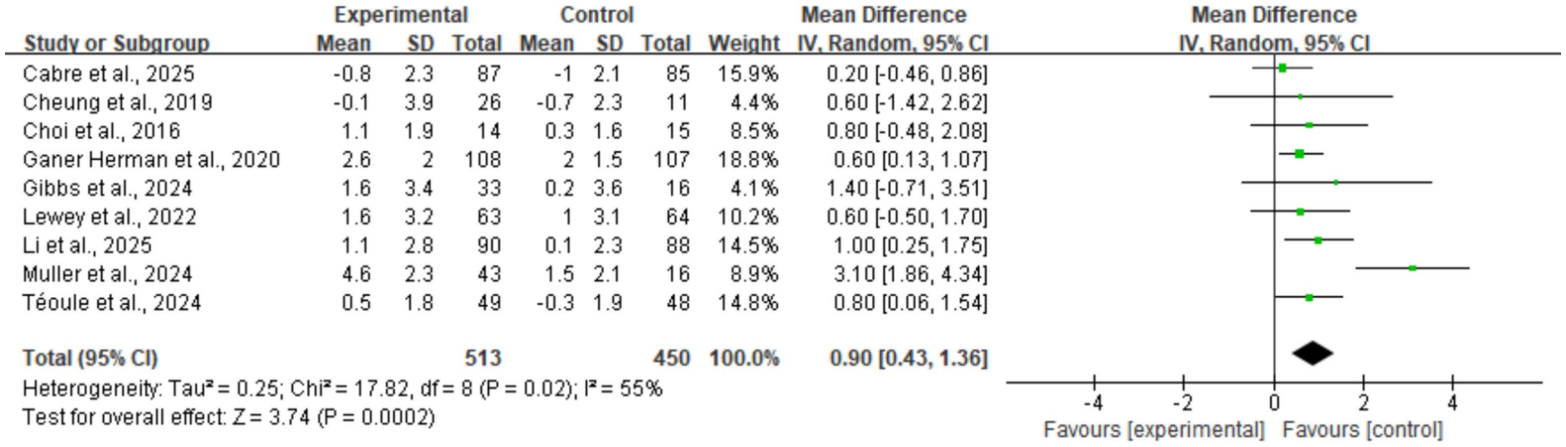
Forest plot of daily step counts.

Data regarding step counts were synthesized from nine randomized controlled trials (Figure 4). The meta-analysis indicated that digital health interventions significantly increased daily step counts in perinatal women (MD = 0.90, 95% CI: 0.43, 1.36). Statistical testing demonstrated a significant difference (*p* = 0.0002). The test for heterogeneity revealed moderate heterogeneity across studies (I^2^ = 55%). The overall effect diamond was positioned entirely to the right of the line of no effect, confirming the efficacy of the interventions in increasing step counts.

Following the exclusion of Muller et al. (30)—a study assessed as having a “high risk” of bias—heterogeneity for the step count metric dropped significantly from 55 to 0%, verifying this study as the primary source of the observed heterogeneity. The sensitivity analysis yielded an adjusted pooled mean difference of 0.68 (95% CI, 0.44, 0.92), with statistical significance remaining extremely high (*p* < 0.00001). These findings demonstrate that, after excluding extreme outliers, digital health interventions consistently and significantly increased daily activity by approximately 680 steps, reflecting high robustness of the results.

#### 3.4.2 Light physical activity (LPA)

**Figure 5:**
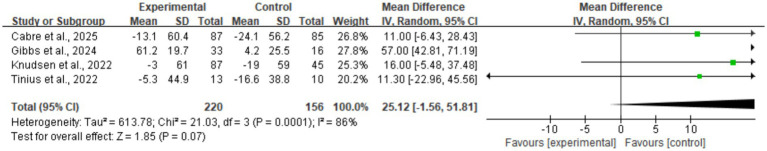
Forest plot of light physical activity (LPA).

Four studies were included for the LPA outcome (Figure 5). The pooled effect size showed a mean increase of 25.12 min/day in the intervention group compared to the control group (MD = 25.12, 95% CI: −1.56, 51.81). However, the difference did not reach statistical significance (*p* = 0.07). The heterogeneity test indicated extremely high heterogeneity (*I*^2^ = 86%), which was primarily driven by the wide span of effect sizes across the different studies.

In the sensitivity analysis for LPA, utilizing the leave-one-out method to exclude Gibbs et al. (32) resulted in a significant shift in the pooled outcome. Inter-study heterogeneity decreased substantially from 86 to 0%, indicating high consistency in the direction of effects among the remaining studies. The adjusted pooled mean difference (MD) was 13.04 min/day (95% CI, 1.15, 24.92), and the statistical test reached significance (*p* = 0.03). Contrasted with the borderline significance observed prior to exclusion (*p* = 0.07), the sensitivity analysis confirmed that digital health interventions exert a stable and significant promoting effect on light physical activity in perinatal women.

#### 3.4.3 Moderate-to-vigorous physical activity (MVPA)

**Figure 6:**
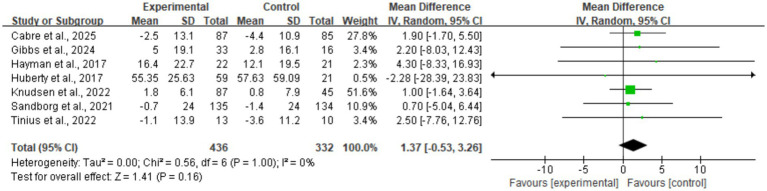
Forest plot of moderate-to-vigorous physical activity (MVPA).

Data for MVPA were pooled from seven studies (Figure 6), yielding a mean difference of 1.37 min/day (MD = 1.37, 95% CI: −0.53, 3.26). The result was not statistically significant (*p* = 0.16). The heterogeneity test indicated excellent consistency across studies (*I*^2^ = 0%), suggesting an absence of clinical heterogeneity. This reflects that current digital health strategies generally produce a consistent but weak effect on engaging participants in moderate-to-vigorous physical activity. Sensitivity analysis showed no directional change in the pooled MVPA results, and the 95% CI consistently included zero, indicating that the findings are robust and not driven by any single study.

#### 3.4.4 Sedentary behavior

**Figure 7:**
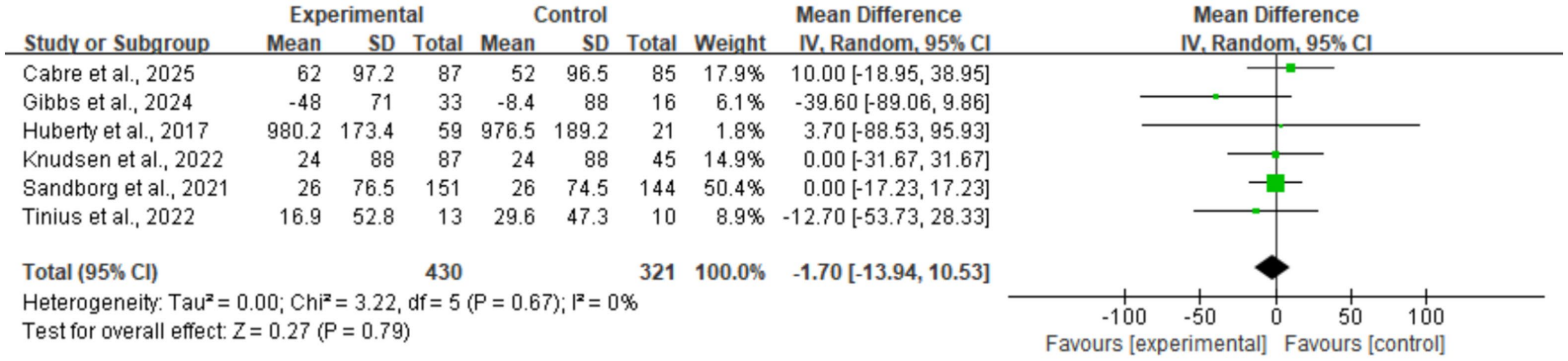
Forest plot of sedentary time.

Data on sedentary time were synthesized from six studies (Figure 7). The meta-analysis revealed almost no difference between the intervention and control groups (MD = −1.70, 95% CI: −13.94, 10.53). Statistical testing showed the result was non-significant (*p* = 0.79). No significant heterogeneity was observed between studies (*I*^2^ = 0%). These results suggest that existing digital health interventions have not effectively ameliorated sedentary habits among perinatal women. Sensitivity analysis demonstrated that the results for sedentary time remained stable (*p* > 0.05) regardless of the exclusion of any individual study, further confirming the non-significant effect of the interventions based on current evidence.

### 3.5 Subgroup analyses

**Figure 8:**
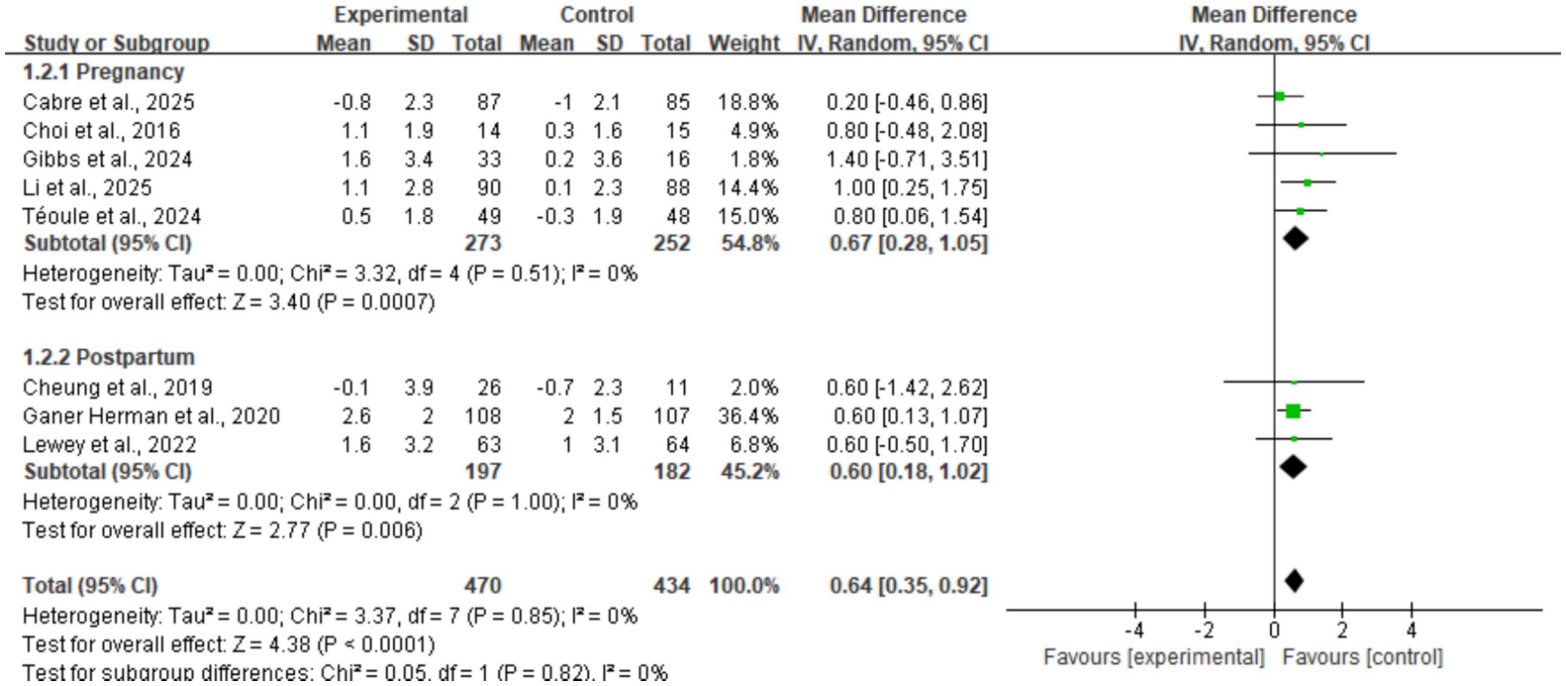
Subgroup forest plot of daily step counts stratified by study phase.

Given the moderate heterogeneity initially observed in the step count metric (*I*^2^ = 55%) and the identification of Muller et al. (30)—a study with a high risk of bias—as the primary source of this heterogeneity via sensitivity analysis, a subgroup analysis based on the study phase (pregnancy vs. postpartum) was conducted after excluding this study (Figure 8). Following exclusion, the heterogeneity of the overall effect size decreased to *I*^2^ = 0%, significantly enhancing the certainty of the results. The subgroup analysis revealed that mHealth interventions significantly increased daily step counts in the pregnancy subgroup (*n* = 5; MD = 0.67, 95% CI: 0.28, 1.05, *p* < 0.001). Similarly, a consistent positive effect was observed in the postpartum subgroup (*n* = 3; MD = 0.60, 95% CI: 0.18, 1.02, *p* = 0.006). The test for subgroup differences indicated no statistical significance ($*p* = 0.82, *I*^2^ = 0%), suggesting that the efficacy of mHealth interventions in promoting physical activity remains highly consistent across different stages of the perinatal period.

**Figure 9:**
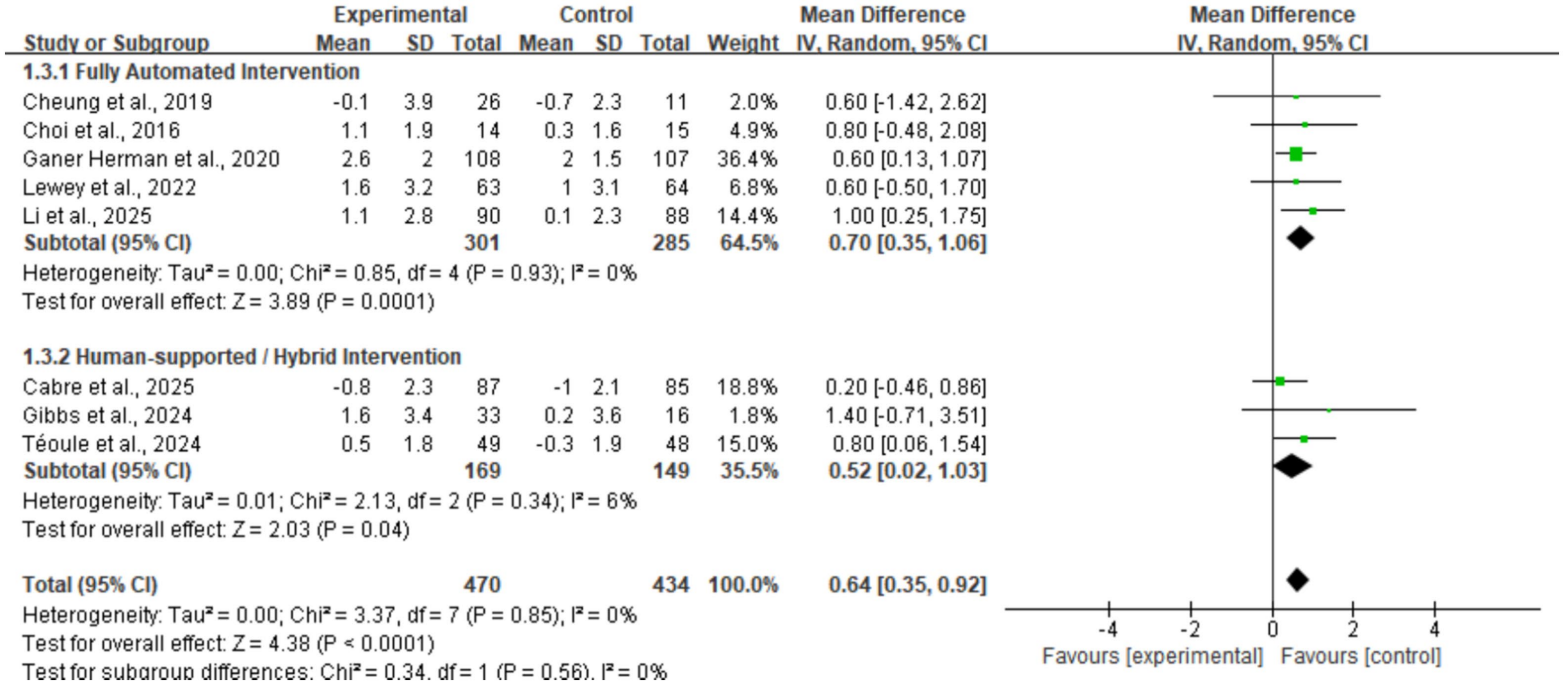
Subgroup forest plot of daily step counts stratified by intervention delivery mode.

Subgroup analysis regarding intervention delivery modes (Figure 9) demonstrated that both fully automated interventions (*n* = 5) and human-assisted/hybrid interventions (*n* = 3) significantly increased daily step counts in perinatal women, with effect sizes (MD) of 0.70 (95% CI: 0.35, 1.06, *p* = 0.0001) and 0.52 (95% CI: 0.02, 1.03, *p* = 0.04), respectively. The test for subgroup differences showed no statistical significance (*p* = 0.56, *I*^2^ = 0%), indicating that the effectiveness of mHealth interventions is not dependent on the inclusion of human support components.

**Figure 10:**
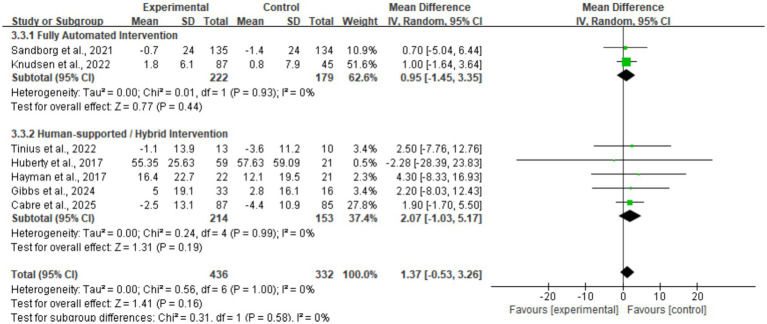
Subgroup forest plot of MVPA stratified by intervention delivery mode.

Regarding MVPA (Figure 10), subgroup analysis indicated that the mode of intervention delivery did not significantly moderate intervention effects. Neither fully automated interventions (MD = 0.95, 95% CI: −1.45, 3.35, *p* = 0.44) nor human-assisted/hybrid interventions (MD = 2.07, 95% CI: −1.03, 5.17, *p* = 0.19) yielded statistically significant results. The test for subgroup differences yielded *p* = 0.58 and *I*^2^ = 0%, suggesting that adding human support components failed to significantly enhance the efficacy of mHealth interventions for MVPA. Heterogeneity within all subgroups was 0%, reaffirming the high consistency of findings for this metric.

**Figure 11:**
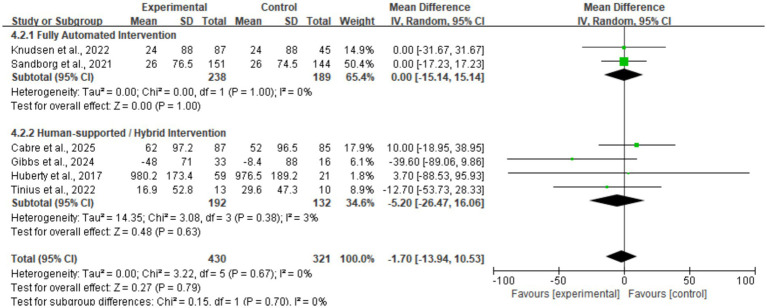
Subgroup forest plot of sedentary time stratified by intervention delivery mode.

Subgroup analysis of sedentary time by delivery mode (Figure 11) revealed that intervention effects were non-significant regardless of the technological delivery method employed. Specifically, the fully automated intervention subgroup (*n* = 2) showed a pooled mean difference of 0.00 min/day (95% CI: −15.14, 15.14, *p* = 1.00), indicating absolutely no difference between the intervention and control groups. Although the human-assisted/hybrid intervention subgroup (*n* = 4) exhibited a slight decreasing trend (MD = −5.20, 95% CI: −26.47, 16.06), the statistical test remained non-significant (*p* = 0.63). The test for subgroup differences (*p* = 0.70, *I*^2^ = 0%) indicates that the addition of human support components did not significantly improve the potency of mHealth interventions in reducing sedentary behavior.

### 3.6 Assessment of publication bias

This study included an *a priori* plan to assess publication bias using funnel plots and Egger’s test. However, in accordance with the recommendations of the Cochrane Handbook (35), tests for funnel plot asymmetry may lack sufficient statistical power to yield reliable judgments when a meta-analysis includes fewer than 10 studies. Consequently, as the number of included studies for each outcome indicator (daily steps, LPA, MVPA, and sedentary time) fell below this threshold, no statistical assessment of publication bias was performed.

### 3.7 Certainty of evidence

The certainty of the evidence was evaluated using the GRADE system (Table 2). The quality of evidence for daily step counts was rated as “moderate,” with downgrading primarily attributed to the risk of bias arising from the lack of blinding. Crucially, this lack of blinding is considered an intrinsic limitation of behavioral physical activity interventions, where blinding participants and personnel to the intervention allocation is generally unfeasible, rather than a flaw in study design. The quality of evidence for LPA, MVPA, and sedentary time was rated as “low”; in addition to the aforementioned inherent blinding constraints, these outcomes were downgraded for imprecision arising from small sample sizes and non-significant pooled estimates. Notably, all outcome measures demonstrated extremely high consistency (*I*^2^ = 0%), attesting to the robustness of the findings.

**Table 2:** GRADE evidence quality evaluation.

Outcome	No. of studies (*N*)	Risk of bias	Inconsistency	Indirectness	Imprecision	Publication bias	Certainty level
Daily steps	8 (904)	Serious	Not serious	Not serious	Not serious	Not assessed	Moderate
Light physical activity (LPA)	3 (327)	Serious	Not serious	Not serious	Serious	Not assessed	Low
Moderate-to-vigorous physical activity (MVPA)	7 (768)	Serious	Not serious	Not serious	Serious	Not assessed	Low
Sedentary time	6 (751)	Serious	Not serious	Not serious	Serious	Not assessed	Low

## 4 Discussion

### 4.1 Principal findings

This systematic review and meta-analysis included 14 randomized controlled trials involving 2,101 participants to systematically evaluate the effects of digital health interventions on objectively measured physical activity and sedentary behavior in perinatal women. The overall findings indicate that digital health interventions are significantly effective in increasing daily step counts and light physical activity (LPA) among perinatal women; however, they did not yield significant improvements in moderate-to-vigorous physical activity (MVPA) or the reduction of sedentary time.

Daily step count emerged as the outcome with the highest quality of evidence and the most robust results in this study. Following the exclusion of studies with a high risk of bias, digital health interventions elicited a significant mean increase of approximately 680 steps per day in perinatal women, with inter-study heterogeneity reduced to 0%. Subgroup analyses further demonstrated that this positive effect remained highly consistent across different perinatal stages and various technology delivery modes. This suggests that digital health interventions, as a convenient modality, possess generalizability in enhancing baseline daily activity levels among perinatal women. Regarding light physical activity (LPA), sensitivity analysis similarly confirmed a significant promoting effect of digital health interventions, with the intervention group showing an average daily increase of approximately 13 min.

Conversely, this study found that improvements in MVPA and sedentary time were non-significant, with extremely high consistency observed across studies (*I*^2^ = 0%). Even within subgroups incorporating human support or video coaching, these two metrics failed to achieve statistical significance. These findings suggest that while current digital health strategies can effectively “drive” perinatal women to increase daily low-intensity activities such as walking, they face substantial challenges in overcoming physiological or environmental barriers to induce higher-intensity exercise and in breaking established sedentary habits. Overall, digital health interventions demonstrate clear preliminary efficacy in perinatal exercise management; however, future research must optimize intervention designs to achieve more profound behavioral changes.

### 4.2 Comparison with previous studies and mechanism analysis

This study, by synthesizing data from the 14 included RCTs, found that digital health interventions significantly increased daily step counts and light physical activity (LPA) in perinatal women. These findings largely validate and extend previous conclusions regarding the efficacy of digital technologies in promoting physical activity (12). Compared to prior reviews that relied heavily on self-reported data, the conclusions of this study, derived from objective measurements, demonstrate a higher degree of certainty (14). The extremely low heterogeneity observed in the step count metric provides compelling evidence for the generalizability of digital health interventions in enhancing daily activity volume. However, the non-significant results regarding MVPA and sedentary time differ from the positive trends observed in some general population studies (36). This discrepancy likely reflects the unique characteristics of the perinatal population; constrained by physiological burdens such as pregnancy-related fatigue, postpartum physical recovery, and childcare demands (37), simple digital “nudges” are more likely to induce low-intensity behaviors like walking rather than overcoming barriers to high-intensity exercise or disrupting long-established sedentary patterns (38).

Through sensitivity and subgroup analyses, this study conducted an in-depth investigation into the sources of heterogeneity, revealing key factors influencing intervention efficacy. Regarding LPA, heterogeneity dropped from 86 to 0% after excluding Gibbs et al. (32). The markedly larger effect size in that study was primarily driven by the introduction of “standing desks”—a physical environmental restructuring technique—whereas other studies remained limited to purely digital reminders. This finding holds significant practical implications, suggesting that integrating physical environmental changes with digital interventions may be a primary direction for improving low-intensity activity in the future. Furthermore, subgroup analysis for step counts indicated that fully automated interventions yielded slightly superior effects compared to human-assisted interventions. This suggests that during the perinatal period, a phase characterized by high time costs, immediate and low-interference digital feedback (such as real-time App synchronization and automated push notifications) may align better with women’s fragmented lifestyles than appointment-based human counseling, highlighting the superiority of low-cost digital solutions in public health management (39).

From a theoretical perspective, the variation in performance across different indicators can be explained by the depth of engagement required by Behavior Change Techniques (BCTs) (40). The included studies widely integrated core BCTs such as “goal setting,” “self-monitoring,” and “biofeedback.” These techniques exert a strong regulatory effect on low-barrier, accessible daily activities like walking (Steps/LPA) and effectively enhance individual compliance (41, 42). However, increasing MVPA typically necessitates deeper technical support, such as “demonstration of behavior,” “problem-solving,” and “safety guidance” (43). In the perinatal context, women often harbor inherent safety concerns regarding high-intensity exercise and face physical bottlenecks (44); consequently, current digital health protocols lacking targeted physiological assessments and personalized exercise prescriptions struggle to produce statistically significant qualitative changes. Similarly, sedentary behavior in this population is often deeply intertwined with childcare tasks, such as breastfeeding and rest, making it highly resistant to change (45). The consistent non-significance of the sedentary metric in this study suggests that future digital health designs should shift from simple “exercise promotion” to targeted “sedentary interruption” interventions, optimized to accommodate the specific behavioral patterns of the perinatal period.

### 4.3 Practical implications

The findings of this study—that digital health interventions consistently increase daily step counts and light physical activity (LPA) in perinatal women—provide a clear evidence base for clinical perinatal care. Perinatal women often struggle to adhere to high-intensity fitness regimens due to fragmented time, parenting stress, and physiological changes (25). Furthermore, psychological barriers, including conservative health beliefs and fear regarding the safety of vigorous exercise during pregnancy, often lead to behavioral inhibition. In contrast, digital health protocols based on smartphone applications or wearable devices demonstrate superior participant adherence due to their low barriers to entry, real-time feedback capabilities, and high accessibility (33). Crucially, these tools can bridge the “intention-behavior gap” by providing evidence-based safety guidance and continuous psychological reinforcement, which helps to reshape maternal health beliefs and alleviate anxieties related to physical exertion. Notably, although the observed increments of 680 steps per day and 13 min per day in LPA may appear modest in absolute magnitude, they could potentially represent a “minimum effective dose” necessary to elicit improvements in perinatal metabolic health. During this metabolically sensitive window, such subtle shifts from sedentary behavior toward light activity might be sufficient to enhance glucose metabolism or mitigate excessive gestational weight gain (46), possessing foundational public health value for preventing gestational obesity and assisting in the management of gestational diabetes mellitus. Given the advantages of low entry barriers and high adherence, clinical prenatal and postpartum care should integrate these tools into routine health management systems as a “minimum effective dose” intervention for maintaining maternal baseline metabolic health.

However, the non-significant results regarding moderate-to-vigorous physical activity (MVPA) and sedentary behavior reveal the efficacy boundaries of current standalone digital interventions. While existing technologies can effectively drive low-intensity daily activities, they are evidently insufficient to assist participants in overcoming the physiological and psychological barriers to high-intensity exercise; the actual increments remain substantially below the “150 minutes per week” clinical benefit standard recommended by the WHO (47, 48). This “intensity gap” suggests that for high-risk populations requiring strict weight control or with confirmed complications, reliance solely on digital monitoring and feedback may fail to trigger behavioral modifications sufficient to alter clinical outcomes. Future practice must explore hybrid models that combine digital tools with more coercive environmental restructuring or professional exercise prescriptions.

Furthermore, combining the subgroup analysis finding that “automated delivery and human-assisted modes have comparable effects,” this study supports the implementation of a cost-effective “stepped-care” strategy in resource-limited settings. Specifically, this entails prioritizing the broad promotion of algorithm-based automated digital intervention protocols to the general perinatal population to achieve universal baseline activity improvements at minimal cost. Meanwhile, expensive human guidance resources should be precisely reserved for specific high-risk subgroups who are non-responders to automated interventions or who need to cross higher intensity thresholds. This evidence-based stratified resource allocation model promises to maximize the coverage advantages of digital technology while significantly enhancing the overall efficacy of perinatal health services.

### 4.4 Limitations

Despite providing objective evidence regarding the impact of digital health interventions on perinatal physical activity, this study is subject to several limitations. First, the number of included studies was relatively limited (14 RCTs in total), with only three studies remaining for the LPA outcome following sensitivity analysis. In accordance with Cochrane Collaboration recommendations, as the number of studies for each outcome was fewer than 10, reliable statistical assessment of publication bias via funnel plots or Egger’s tests was not feasible, which may, to some extent, limit the comprehensiveness of the findings. Furthermore, the certainty of evidence for outcomes such as LPA, MVPA, and sedentary time was graded as “low” due to limited sample sizes or non-significant pooled effects, suggesting that necessary caution should be exercised when interpreting these findings.

Concurrently, this study faced risk-of-bias challenges common to behavioral intervention trials. Due to the inherent nature of physical exercise and digital technology interventions, double-blinding of participants and personnel was not possible in any of the included studies, resulting in a universal classification of “some concerns” in the “deviations from intended interventions” domain of the RoB 2.0 assessment. Additionally, digital health interventions face challenges regarding participant attrition or reduced compliance with objective device wear protocols during long-term implementation; high dropout rates or data loss in some studies may have potentially compromised the precision of the pooled results. Although random-effects models and sensitivity analyses were employed to manage heterogeneity, inherent variations among participants regarding baseline BMI, pregnancy risk profiles, and digital literacy may remain as potential confounding factors influencing intervention efficacy.

Finally, while this study uniformly utilized objective hardware devices for data recording, discrepancies in device brands (e.g., consumer-grade Fitbits vs. clinical-grade ActiGraphs) and wear protocols (e.g., wrist vs. waist placement, valid wear-day requirements) across studies may introduce minor systematic variations in measurement sensitivity. Moreover, this study primarily focused on changes in physical activity behavioral metrics and did not extensively explore the dose–response relationship between digital health interventions and long-term maternal and infant clinical outcomes, such as the incidence of gestational complications or delivery outcomes. Future research warrants more large-scale, multi-center randomized controlled trials focusing on long-term clinical benefits to further substantiate the evidence base for digital health in perinatal exercise management.

## 5 Conclusion

This systematic review and meta-analysis provides preliminary evidence that digital health interventions represent a potentially effective strategy for improving physical activity among perinatal women. The findings indicate that digital modalities robustly increase daily step counts and light physical activity (LPA), with this positive effect demonstrating high consistency across different perinatal stages and regardless of whether the delivery mode is automated or human-assisted. However, there is currently insufficient evidence to support the efficacy of digital health interventions in effectively enhancing moderate-to-vigorous physical activity (MVPA) or significantly reducing sedentary time, highlighting the limitations of current intervention designs in driving higher-intensity behavioral changes. Future intervention protocols should consider integrating environmental restructuring or utilizing more targeted, personalized exercise prescriptions to achieve the optimal intervention dose necessary for improving perinatal maternal and infant health.

## Data Availability

The original contributions presented in the study are included in the article/Supplementary material, further inquiries can be directed to the corresponding author.
