# Supplementary material for: Effects of digital health interventions on objectively measured physical activity during the perinatal period: a systematic review and meta-analysis
Source: Front Public Health. 2026 Apr 2;14:1786474. doi: 10.3389/fpubh.2026.1786474 (PMC13083171; doi:10.3389/fpubh.2026.1786474)
Supplement: Supplementary file 1 [file Table_1.docx]

Supplementary Text 1 Search Strategies

1. **PubMed**

search strategy:

#1 ("Perinatal Care"[MeSH Terms] OR "Postpartum Period"[MeSH Terms] OR "Pregnancy"[MeSH Terms] OR ("pregnan*"[Title/Abstract] OR "prenatal"[Title/Abstract] OR "antenatal"[Title/Abstract] OR "perinatal"[Title/Abstract] OR "postpartum"[Title/Abstract] OR "post-partum"[Title/Abstract] OR "postnatal"[Title/Abstract] OR "post-natal"[Title/Abstract] OR "gestation*"[Title/Abstract] OR "expectant mother*"[Title/Abstract]))

#2 ("Mobile Applications"[MeSH Terms] OR "Telemedicine"[MeSH Terms] OR "Smartphone"[MeSH Terms] OR "Internet-Based Intervention"[MeSH Terms] OR "Social Media"[MeSH Terms] OR "Wearable Electronic Devices"[MeSH Terms] OR "Fitness Trackers"[MeSH Terms] OR "Text Messaging"[MeSH Terms] OR ("Digital Health"[Title/Abstract] OR "eHealth"[Title/Abstract] OR "mHealth"[Title/Abstract] OR "mobile app*"[Title/Abstract] OR "smartphone app*"[Title/Abstract] OR "web-based"[Title/Abstract] OR "internet-based"[Title/Abstract] OR "online intervention*"[Title/Abstract] OR "text messag*"[Title/Abstract] OR "SMS"[Title/Abstract] OR "wearable*"[Title/Abstract] OR "fitness tracker*"[Title/Abstract] OR "smartwatch*"[Title/Abstract] OR "Social Media"[Title/Abstract] OR "gamif*"[Title/Abstract] OR "exergame*"[Title/Abstract] OR "tele-rehabilitation"[Title/Abstract] OR "telehealth"[Title/Abstract]))

#3 ("Exercise"[MeSH Terms] OR "Motor Activity"[MeSH Terms] OR "Sedentary Behavior"[MeSH Terms] OR "Physical Exertion"[MeSH Terms] OR ("physical activity"[Title/Abstract] OR "exercise*"[Title/Abstract] OR "walking"[Title/Abstract] OR "steps"[Title/Abstract] OR "MVPA"[Title/Abstract] OR "moderate to vigorous physical activity"[Title/Abstract] OR "sedentary time"[Title/Abstract] OR "sitting time"[Title/Abstract] OR "sedentary behavior*"[Title/Abstract] OR "total physical activity"[Title/Abstract] OR "TPA"[Title/Abstract] OR "MET"[Title/Abstract]))

#4 ("randomized controlled trial"[Publication Type] OR "controlled clinical trial"[Publication Type] OR "randomized"[Title/Abstract] OR "placebo"[Title/Abstract] OR "clinical trials as topic"[MeSH Terms:noexp] OR "randomly"[Title/Abstract] OR "trial"[Title])

#5 #1 AND #2 AND #3 AND #4

1. **Web of science**

search strategy:

#1 TS= (Perinatal Care OR Postpartum Period OR Pregnancy OR pregnan OR prenatal OR antenatal OR perinatal OR postpartum OR post-partum OR postnatal OR post-natal OR gestation OR expectant mother)

#2 TS= (Mobile Applications OR Telemedicine OR Smartphone OR Internet-Based Intervention OR Social Media OR Wearable Electronic Devices OR Fitness Trackers OR Text Messaging OR Digital Health OR eHealth OR mHealth OR mobile app OR smartphone app OR web-based OR internet-based OR online intervention OR text messag OR SMS OR wearable OR fitness tracker OR smartwatch OR Social Media OR gamif OR exergame OR tele-rehabilitation OR telehealth)

#3 TS= (Exercise OR Motor Activity OR Sedentary Behavior OR Physical Exertion OR physical activity OR exercise OR walking OR steps OR MVPA OR moderate to vigorous physical activity OR sedentary time OR sitting time OR sedentary behavior OR total physical activity OR TPA OR MET)

#4 TS= (randomized controlled trial OR controlled clinical trial OR randomized OR placebo OR clinical trials as topic OR randomly OR trial)

#5 #1 AND #2 AND #3 AND #4

1. **Embase**

search strategy:

#1 ti,ab,kw= (Perinatal Care OR Postpartum Period OR Pregnancy OR pregnan OR prenatal OR antenatal OR perinatal OR postpartum OR post-partum OR postnatal OR post-natal OR gestation OR expectant mother)

#2 ti,ab,kw= (Mobile Applications OR Telemedicine OR Smartphone OR Internet-Based Intervention OR Social Media OR Wearable Electronic Devices OR Fitness Trackers OR Text Messaging OR Digital Health OR eHealth OR mHealth OR mobile app OR smartphone app OR web-based OR internet-based OR online intervention OR text messag OR SMS OR wearable OR fitness tracker OR smartwatch OR Social Media OR gamif OR exergame OR tele-rehabilitation OR telehealth)

#3 ti,ab,kw= (Exercise OR Motor Activity OR Sedentary Behavior OR Physical Exertion OR physical activity OR exercise OR walking OR steps OR MVPA OR moderate to vigorous physical activity OR sedentary time OR sitting time OR sedentary behavior OR total physical activity OR TPA OR MET)

#4 ti,ab,kw= (randomized controlled trial OR controlled clinical trial OR randomized OR placebo OR clinical trials as topic OR randomly OR trial)

#5 #1 AND #2 AND #3 AND #4

1. **CINAHL**

search strategy:

#1 XB= (Perinatal Care OR Postpartum Period OR Pregnancy OR pregnan OR prenatal OR antenatal OR perinatal OR postpartum OR post-partum OR postnatal OR post-natal OR gestation OR expectant mother)

#2 XB= (Mobile Applications OR Telemedicine OR Smartphone OR Internet-Based Intervention OR Social Media OR Wearable Electronic Devices OR Fitness Trackers OR Text Messaging OR Digital Health OR eHealth OR mHealth OR mobile app OR smartphone app OR web-based OR internet-based OR online intervention OR text messag OR SMS OR wearable OR fitness tracker OR smartwatch OR Social Media OR gamif OR exergame OR tele-rehabilitation OR telehealth)

#3 XB= (Exercise OR Motor Activity OR Sedentary Behavior OR Physical Exertion OR physical activity OR exercise OR walking OR steps OR MVPA OR moderate to vigorous physical activity OR sedentary time OR sitting time OR sedentary behavior OR total physical activity OR TPA OR MET)

#4 XB= (randomized controlled trial OR controlled clinical trial OR randomized OR placebo OR clinical trials as topic OR randomly OR trial)

#5 #1 AND #2 AND #3 AND #4

1. **Cochrane Library**

search strategy:

#1 ti,ab,kw= (Perinatal Care OR Postpartum Period OR Pregnancy OR pregnan OR prenatal OR antenatal OR perinatal OR postpartum OR post-partum OR postnatal OR post-natal OR gestation OR expectant mother)

#2 ti,ab,kw= (Mobile Applications OR Telemedicine OR Smartphone OR Internet-Based Intervention OR Social Media OR Wearable Electronic Devices OR Fitness Trackers OR Text Messaging OR Digital Health OR eHealth OR mHealth OR mobile app OR smartphone app OR web-based OR internet-based OR online intervention OR text messag OR SMS OR wearable OR fitness tracker OR smartwatch OR Social Media OR gamif OR exergame OR tele-rehabilitation OR telehealth)

#3 ti,ab,kw= (Exercise OR Motor Activity OR Sedentary Behavior OR Physical Exertion OR physical activity OR exercise OR walking OR steps OR MVPA OR moderate to vigorous physical activity OR sedentary time OR sitting time OR sedentary behavior OR total physical activity OR TPA OR MET)

#4 ti,ab,kw= (randomized controlled trial OR controlled clinical trial OR randomized OR placebo OR clinical trials as topic OR randomly OR trial)

#5 #1 AND #2 AND #3 AND #4
